# Predicted climate change will increase the truffle cultivation potential in central Europe

**DOI:** 10.1038/s41598-020-76177-0

**Published:** 2020-12-04

**Authors:** Tomáš Čejka, Miroslav Trnka, Paul J. Krusic, Ulrich Stobbe, Daniel Oliach, Tomáš Václavík, Willy Tegel, Ulf Büntgen

**Affiliations:** 1 Department of Climate Change Impacts On Agroecosystems, Global Change Research Institute of the Czech Academy of Sciences, Bělidla 986/4, 603 00 Brno, Czech Republic; 2 Department of Geography, Faculty of Science, Masaryk University, Kotlářská 2, 602 00 Brno, Czech Republic; 3 Department of Agrosystems and Bioclimatology, Faculty of AgriSciences, Mendel University, Zemědělská 1, 613 00 Brno, Czech Republic; 4 Department of Geography, University of Cambridge, Downing Place, Cambridge, CB2 3EN UK; 5 Department of Physical Geography, Stockholm University, 106 91, Stockholm, Sweden; 6 Deutsche Trüffelbäume, Karl-Bücheler Strasse 1, 78315 Radolfzell, Germany; 7 Forest Science and Technology Centre of Catalonia (CTFC), Crta. Sant Llorenç de Morunys km 2, 25280 Solsona, Spain; 8 Department of Crop and Forest Science, University of Lleida, Alcalde Rovira Roure 191, 25198 Lleida, Spain; 9 Department of Ecology and Environmental Sciences, Palacký University, Šlechtitelů 27, 78371 Olomouc, Czech Republic; 10 Institute of Forest Sciences, Chair of Forest Growth and Dendroecology, University of Freiburg, Tennenbacher Straße 4, 79106 Freiburg, Germany; 11 Swiss Federal Institute for Forest, Snow and Landscape Research WSL, Zürcherstrasse 111, 8903 Birmensdorf, Switzerland

**Keywords:** Climate-change impacts, Climate-change mitigation, Projection and prediction, Agroecology, Biodiversity, Climate-change ecology, Conservation biology, Ecological modelling, Ecosystem ecology, Ecosystem services, Agroecology, Biogeography, Climate-change ecology, Conservation biology, Ecological modelling, Ecosystem ecology, Ecosystem services

## Abstract

Climate change affects the distribution of many species, including Burgundy and Périgord truffles in central and southern Europe, respectively. The cultivation potential of these high-prized cash crops under future warming, however, remains highly uncertain. Here we perform a literature review to define the ecological requirements for the growth of both truffle species. This information is used to develop niche models, and to estimate their cultivation potential in the Czech Republic under current (2020) and future (2050) climate conditions. The Burgundy truffle is already highly suitable for cultivation on ~ 14% of agricultural land in the Czech Republic (8486 km^2^), whereas only ~ 8% of the warmest part of southern Moravia are currently characterised by a low suitability for Périgord truffles (6418 km^2^). Though rising temperatures under RCP8.5 will reduce the highly suitable cultivation areas by 7%, the 250 km^2^ (3%) expansion under low-emission scenarios will stimulate Burgundy truffles to benefit from future warming. Doubling the moderate and expanding the highly suitable land by 352 km^2^ in 2050, the overall cultivation potential for Périgord truffles will rise substantially. Our findings suggest that Burgundy and Périgord truffles could become important high-value crops for many regions in central Europe with alkaline soils. Although associated with uncertainty, long-term investments in truffle cultivation could generate a wide range of ecological and economic benefits.

## Introduction

Anthropogenic climate change is affecting the elevational and meridional distribution of many ectomycorrhizal fungi^1–4^, which are essential components in natural ecosystems and agriculture^5^. Shifts to higher latitudes and elevations will likely continue in many regions^6^, resulting in either the expansion or contraction of species-specific habitats^7^. Well-informed ecological models can estimate the potential geographic distribution of individual species under future climate change^8,9^.

Predicting how species distributions might change in the future is often based on species distribution models (SDMs), also known as climate envelope models, habitat suitability models, or niche models^10^. Simple correlative SDMs combine information of a species’ current location with abiotic variables, such as climate, soil, and elevation, to predict the probability of a species’ occurrence in space and time^11^. Over the past two decades, more sophisticated SDM techniques, such as random forest^12^ and maximum entropy^13^, as well as population-based (mechanistic) models, dynamic range models, or combinations thereof^11^, have been developed^9^. Although the ‘correlative’ SDM approach is simplistic and excludes certain ecological concepts, such as ‘living dead’ populations and ‘source-sink’ dynamics^11^, it is apparently the most feasible avenue of building niche models for the majority of species^14^, including fungi^15–18^. Similarly, environmental information can be extracted from a rich body of literature to parametrise knowledge-based niche models.

Here, we focus on the Burgundy truffle (*Tuber aestivum* Vittad.) and Périgord truffle (*Tuber melanosporum* Vittad.). Although mainly growing under different ecological conditions, both genera are ectomycorrhizal fungi that live in a symbiotic relationship with their plant partners in the temperate and Mediterranean climates of Europe, Asia, Australia, as well as North and South America^19^. Currently growing in much of Europe^20^, the Burgundy truffle is expected to offer great potential to be cultivated in new regions as climate change progresses^21^. The Périgord truffle has a much smaller ecological range^22^, which restricts its current distribution predominantly to the Mediterranean climate zones of Spain, France, and Italy^23^. The migration of Périgord truffles into higher latitudes north of the European Alps^24,25^, and the recently documented harvest decline in the species’ southern European habitats^26^, have been attributed to climate warming^27^.

Since truffles are commonly exceeding retail prices of ~ 200 € kg^−1^ (Burgundy) and ~ 600 € kg^−1^ (Périgord), their cultivation is economically highly attractive^23,28^. The most productive habitats for the Périgord truffle are natural forests and plantations in southern Europe^23,28^. The cultivation of Périgord truffles is therefore a lucrative business in many parts of rural Spain, France, and Italy^29,30^, whereas the Burgundy truffle is often collected in its natural habitats of temperate and continental Europe^21^. As a result of the increasing gastronomic popularity of truffles, numerous plantations have been established recently in central Europe^20^. Truffle cultivation has many socio-economic and ecological benefits including myco-tourism, increased land value, habitat conservation, and land-use diversification^23,28,30^.

In this study, we review the ecological requirements of Burgundy and Périgord truffles in the existing body of literature, model the current (2020) and future (2050) cultivation potential of both truffle species in the Czech Republic, discuss the uncertainties of our model approach, and outline the implications associated with a possible increase of truffle cultivation in central Europe under future climate change.

## Materials and methods

### Study area and environmental data

To identify those areas that are potentially suitable for the cultivation of truffles under current and predicted climate in 2020 and 2050, respectively, we confined our study to 49–51°N and 12–19°E. This area, within the border of the Czech Republic, contains most of central Europe’s biogeographic zones, and a wide range of geological bedrock. Since truffle growth and maturation require high pH levels^31^, the most suitable bedrock is high-calcium Palaeozoic limestone karst (e.g., Czech Karst, Moravian Karst and Pavlov Hills Mts.), secondary and tertiary deposits^32^ (e.g., Czech Cretaceous basin), as well as Quaternary sediments^33^ (e.g., southern Moravia). The most favourable soils are fertile chernozems and phaeozems below 300–400 m a.s.l., and calcareous leptosols^34^.

Climate zones in the Czech Republic range from temperate maritime in the west to more continental in the east^35^. Based on the 30-year average from 1989–2018, the mean annual temperature varies from 1.7 °C in the mountainous areas (January: − 5.9 °C, July: 10.3 °C) to 10.5 °C in the warmest and driest lowlands (January: − 0.3 °C, July: 20.8 °C). Annual precipitation totals are largely affected by topography and range from 450 mm in the Czech lowlands to 1550 mm at higher elevations in the northeast. Overall, annual precipitation totals reach their maxima in summer (185–505 mm) and minima in winter (60–425 mm). These temperate climate conditions favour a predominance of broad-leaved deciduous forests^34^. However, persistent sylvicultural and agricultural practices over many centuries have resulted in a heterogeneous landscape. The Czech Republic comprises a mosaic of forests and arable land (33.4% and 57.0%, respectively), with the remainder consisting of perennial grasslands and human settlements^34^.

The GIS-based climatological data used to identify suitable areas of truffle growth consist of five raster variables that describe the current ‘baseline climate’ conditions (2020), and five model-derived climate variables for predicting the ‘future climate’ conditions (2050). The 2020 ‘baseline climate’ was defined by both, the mean annual temperature and the temperature extremes of the coldest and warmest months (i.e., January and July). From a network of 268 climatological stations distributed across the Czech Republic, we used precipitation measurements to define the ‘baseline climate’ (1989–2018; Czech Hydrometeorological Institute). With regression kriging that uses altitude as the predictor, we interpolated both, the temperature and precipitation measurements into a 500 × 500 m grid, following common climate resolution standards^36^. To eliminate possible offset from the kriging procedure, we added the delta values to the interpolated layer, thus reduced the modelling error at each station location to zero. Finally, we aggregated the ‘baseline climate’ into monthly, seasonal, and annual temperature means and precipitation totals. Model estimates of the above-mentioned variables from 2041–2060 were hereafter defined as ‘future climate’ and expressed as the year 2050. For the low- (2.6), mid- (4.5), and high-emission (8.5) Representative Concentration Pathways scenarios (RCPs), we computed the combined average of five Global Climate Models (GCMs), i.e. BNU-ESM^37^, CNRM-CM5^38^, HadGEM2-ES^39^, IPSL-CM5A-MR^40^, MRI-CGCM3^41^, which were pre-selected via the evaluation method introduced by Dubrovský et al.^42^. To obtain the ‘future climate’ values, we used the delta-change approach^43^ and added/subtracted delta values as derived from the GCM’s combined average to the monthly parameters of ‘baseline climate’ at 500 × 500 m resolution.

To create GIS-suitable pH data for the entire Czech Republic, we combined two datasets that were available from the State Land Office (Ministry of Agriculture) and the Forest Management Institute. The first dataset includes ~ 150,000 unevenly distributed pH field measurements, mostly from agricultural land at lower elevations, which roughly translated into one measurement per two cells at 500 × 500 m resolution (Supplementary Figure S1). The second dataset includes thousands of soil survey units for the Czech Republic, which are classified into ~ 100 typological units (Supplementary Table S1 and Figure S2). Each unit represents a specific soil type without pH information^44,45^. Since many areas, including forests, lack pH measurements, we extrapolated the field pH measurements to all typological units over the Czech Republic. We did so by attributing the typological unit to the average pH calculated from the field measurements of the corresponding soil survey units (Supplementary Table S1). Since the pH field measurements for a given typological unit generally exhibit a high standard deviation (average ~ 0.45; Supplementary Table S1), we used the median instead of the mean field pH value. Based on a maximum combined area algorithm (via ArcGIS Pro v. 2.3.0)^46^, we were able to cover 64% of the Czech Republic with soil pH information.

The raster elevation model variable was obtained from the ArcČR 500 v. 3.3 database^47^. This model is based on a high-resolution LIDAR mapping performed by the Surveying and Cadastre Office of the Czech Republic. The original data from 2017 were acquired in 5 × 5 m resolution with a vertical error of 0.3 (open area) and 1 (forest) m, respectively. Since the elevation parameter is an indirect expression of temperature and precipitation, it was also considered as a constant proxy for climate that does not change from 2020 to 2050.

### Truffle ecology and modelling

To model current and future areas suitable for Burgundy and Périgord truffle cultivation (in 2020 and 2050), we extracted and synthesized all the available information about the environmental and ecological requirements of both truffle species from 57 scientific publications (Supplementary Table S2). The literature review focussed on temperature means and precipitation totals, elevational ranges, host species, and levels of soil pH (Supplementary Table S2). Except for the host species, each species-specific requirement comprises a set of extracted numerical values that defines the truffle’s theoretically viable ecological range with a probability range from 0.0001–1% of a normal distribution. Each truffle species’ ecological range is equally divided into five suitability classes attributed with a score from 1–5 representing the least and most optimal conditions^48^. Since both species require alkaline soils^31^, the value range with the pH above 7 was divided into five decimal intervals, each of which corresponds to a class of suitability. The uppermost interval above 7.4 obtained the highest score (5), and the lowest (7.0–7.09) was assigned a score of 1 (Supplementary Table S3).

We used a multicriteria analysis^48^ to attribute each truffle requirement parameter with a weight of relative importance (Supplementary Table S3). This was based on the rank sum method^48^, following the expert judgement found in the literature (Supplementary Table S2). With the highest (first) rank of 0.1842, soil pH is the most common nonclimatic property affecting truffle growth^49^. Since the effect of soil chemistry on the pace of truffle ripening is unknown^50^, we consider pH to stimulate growth regardless of the fungus life cycle. While both species slightly differ in phenology^20,51^, annual climatic parameters (mean temperature and total precipitation) are integrated as second in rank/importance (0.1579) to provide a coarse estimate of the potential distribution^51^. The risk of winter frost is represented by January temperature (0.1316), and the likelihood of summer drought is expressed by July temperature and precipitation (0.1316)^20,52^. Elevation was used as the least important parameter (0.1053). The weighted scores were summarized into final suitability values for truffle cultivation, which were then converted to percentages^53^. When addressing differences in the species-specific current and future cultivation potential, the suitability range was divided equally into five rescaled suitability categories^48^. The resulting distribution maps were created using the Overlay Algebra interface in ArcGIS Pro 2.3.0^46^.

## Results

**Figure 1:**
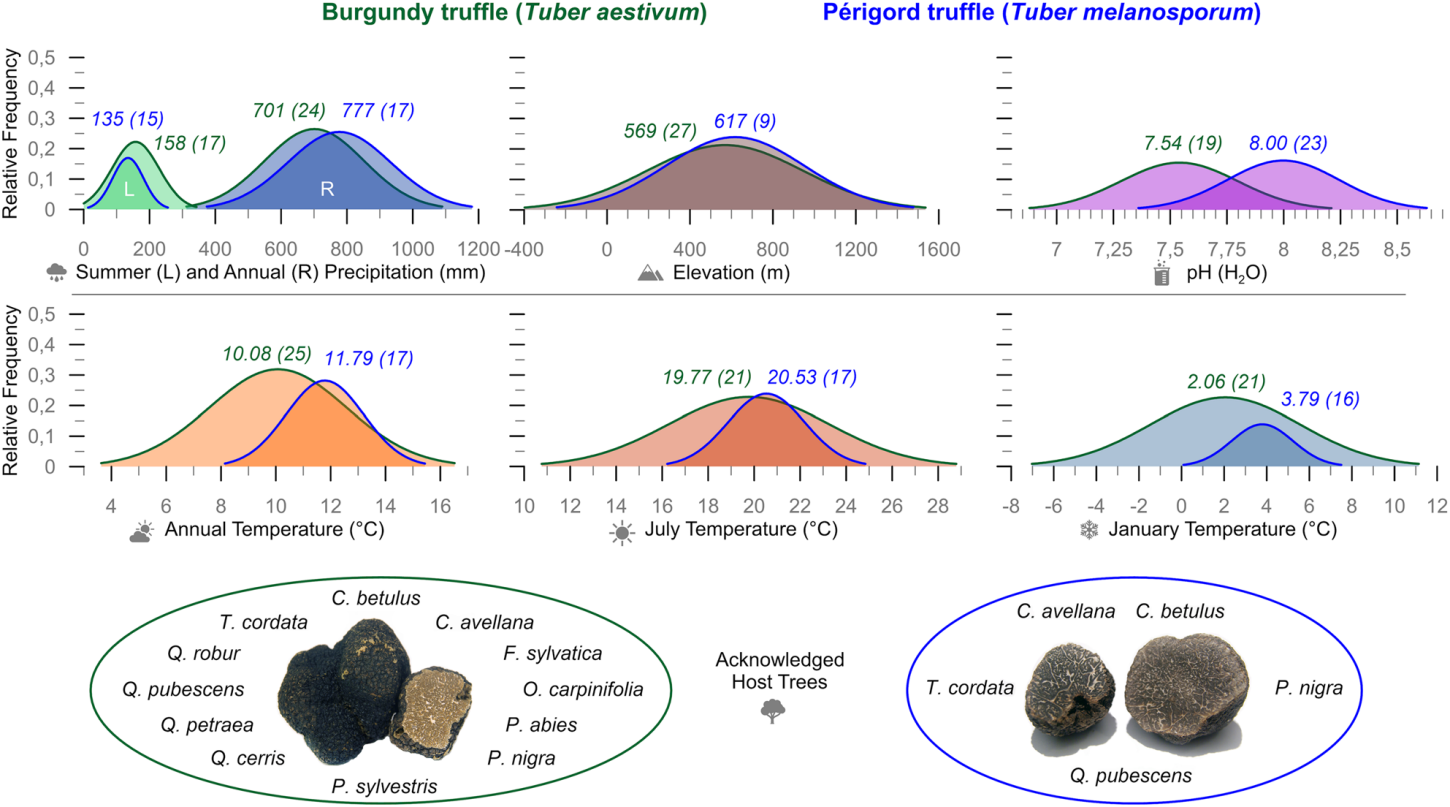
Ecological ranges for Burgundy and Périgord truffle (green and blue line/circle) as probability distribution functions based on numerical values extracted from the literature. Number above each graph represents the mean (optimum) followed by the number of values/studies used for the calculation.

The optimal annual temperature for Burgundy truffle growth is ~ 10 °C, and the ideal July and January temperatures are 19.8 °C and 2.1 °C, respectively (Fig. 1). The Burgundy truffle should receive ~ 700 mm annual precipitation, of which ~ 160 mm should occur in summer. The species’ optimal soil pH is ~ 7.5, and its ideal elevation seems to be ~ 570 m a.s.l.. Favourable host trees for Burgundy truffles in the Czech Republic are *Carpinus betulus*, *Corylus avellana*, *Fagus sylvatica*, *Ostrya carpinifolia*, *Picea abies*, *Pinus nigra*, *Pinus sylvestris*, *Quercus cerris*, *Q. petraea*, *Q. pubescens*, *Q. robur*, and *Tilia cordata*. The optimal annual temperature for Périgord truffle growth is ~ 12 °C, and the ideal July and January temperatures are 20.5 °C and 3.8 °C, respectively (Fig. 1). The Périgord truffle should receive ~ 780 mm of annual precipitation, with at least ~ 140 mm falling in summer. The species favours a pH of ~ 8, and is mainly found at ~ 620 m a.s.l.. Potential host tree species for Périgord truffles in the Czech Republic are *Corylus avellana, Carpinus betulus*, *Pinus nigra, Quercus pubescens,* and *Tilia cordata*. Our results show that Burgundy truffles exhibit a 1.5 to 2.5-fold broader temperature range than Périgord truffles. While Burgundy truffles are 50% more tolerant to changes in summer precipitation, both species exhibit equally broad rainfall requirements. Moreover, both species grow at similar elevations and tolerate similar pH levels.

**Figure 2:**
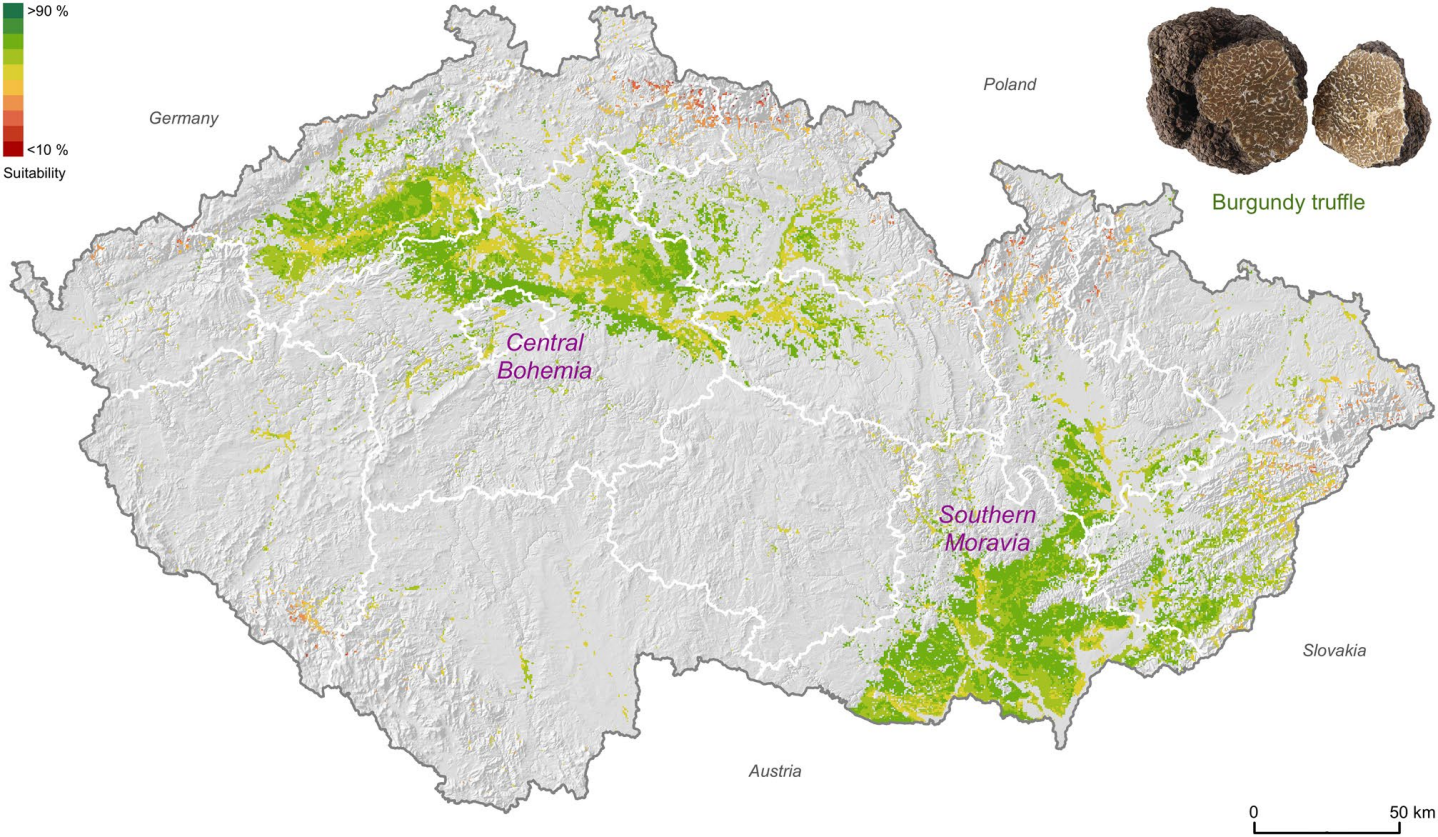
Quantified modelled potential distribution range of suitable Burgundy truffle sites based on pH level, elevational ranges, temperature means, and precipitation totals distinctive for the current climate conditions. The white polygons show the administrative regions of the Czech Republic. The map was created using ArcGIS Pro v. 2.3.0^46^ (https://www.esri.com/en-us/arcgis/products/arcgis-pro/overview).

**Figure 3:**
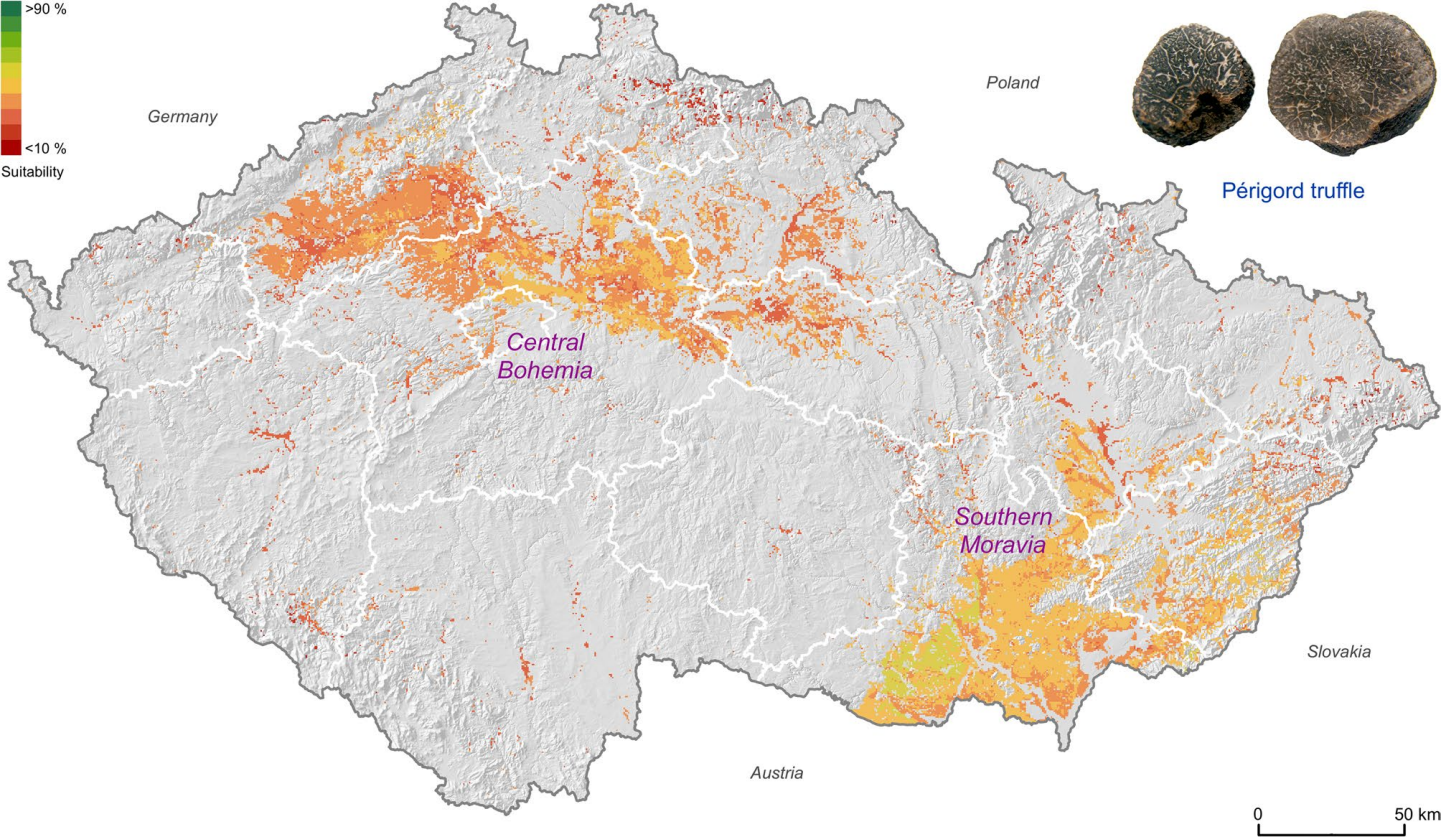
Quantified modelled potential distribution range of suitable Périgord truffle sites based on pH level, elevational ranges, temperature means, and precipitation totals distinctive for the current climate conditions. The white polygons show the administrative regions of the Czech Republic. The map was created using ArcGIS Pro v. 2.3.0^46^ (https://www.esri.com/en-us/arcgis/products/arcgis-pro/overview).

Under the current climate conditions (2020), about 14% of the Czech Republic (77% of the cultivation area) is already highly suitable for the cultivation of Burgundy truffles (8486 km^2^; Table 1). Central Bohemia and southern Moravia exhibit the highest cultivation potential with values between 50 and 80%, respectively (Fig. 2). For the Périgord truffle cultivation potential under current climate conditions, most of the Czech Republic is recognized as low (~ 8%; 6418 km^2^) and moderate (~ 6%; 4482 km^2^) (Table 1). With 30% to 50%, southern Moravia exhibits the highest Périgord truffle cultivation potential (Fig. 3).

**Table 1:** The potentially cultivatable area (km^2^) for categories of suitability for Burgundy truffle and Périgord truffle under current and predicted climate conditions (RCPs) in Czech Republic (78,865 km^2^).

	Suitability in km^2^ ( ±)	Current (2020)	RCP2.6 (2050)	RCP4.5 (2050)	RCP8.5 (2050)
Burgundy truffle	Very low	5	1 (− 4)	1 (− 4)	1 (− 4)
	Low	233	97 (− 136)	63 (− 170)	24 (− 209)
	Moderate	2255	2102 (− 153)	2371 (+ 116)	2884 (+ 629)
	High	8486	8736 (+ 250)	8447 (− 39)	7914 (− 572)
	Very high	2	141 (+ 139)	197 (+ 195)	101 (+ 99)
	Total	10,980	11,077 (+ 97)	11,078 (+ 98)	10,923 (− 57)
Périgord truffle	Very low	109	53 (− 56)	48 (− 61)	27 (− 82)
	Low	6488	2154 (− 4334)	1377 (− 5111)	1175 (− 5313)
	Moderate	4482	8866 (+ 4384)	9559 (+ 5077)	9525 (+ 5043)
	High	0	5 (+ 5)	96 (+ 96)	352 (+ 352)
	Very high	0	0 (0)	0 (0)	0 (0)
	Total	11,079	11,078 (− 1)	11,079 (0)	11,079 (0)

The gain/loss in brackets displays the predicted increase/decrease of area (km^2^) for each category, respectively.

**Figure 4:**
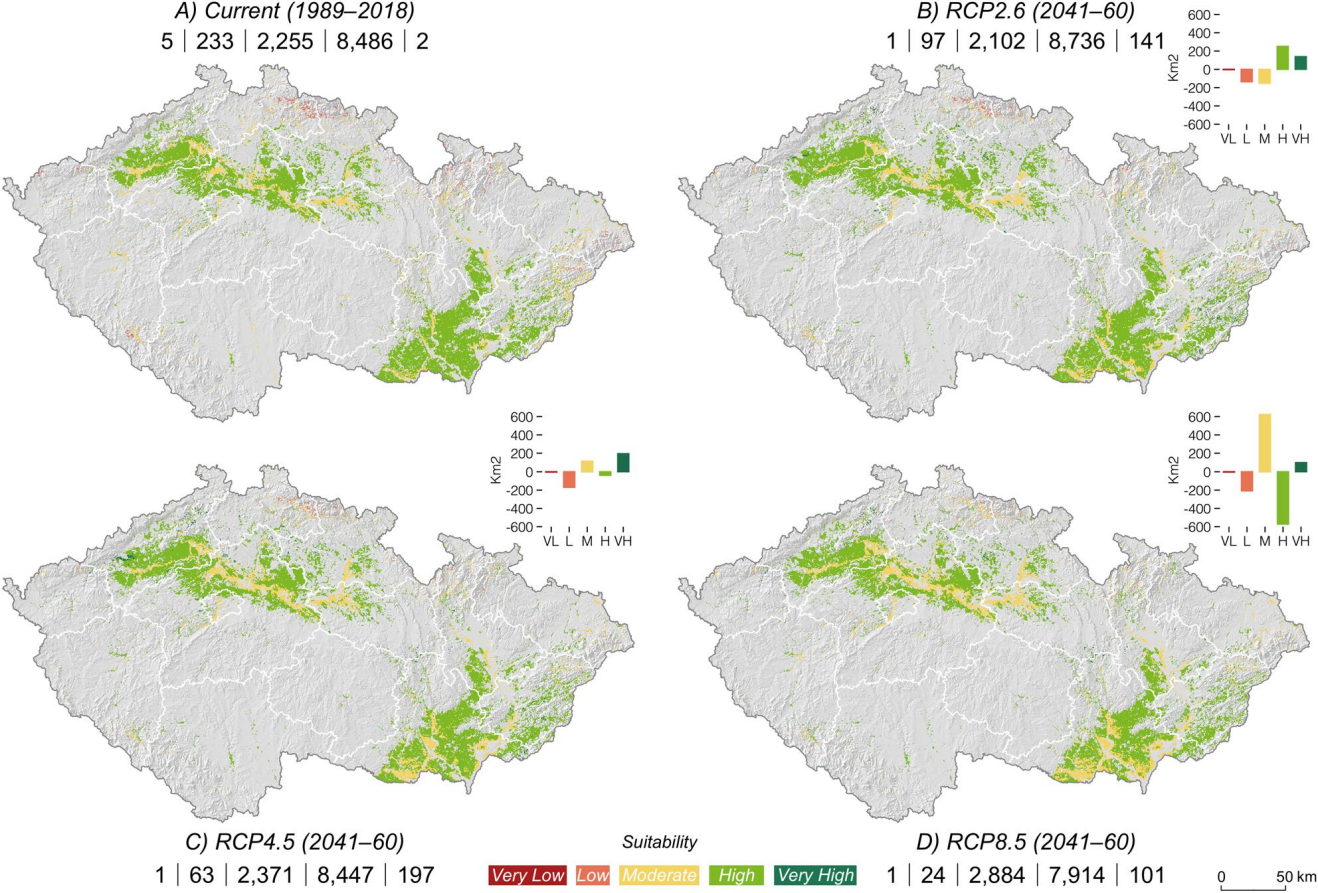
Change of the suitability potential of Burgundy truffle areas in Czech Republic between current (**a**; 1989–2018) and future climate conditions described by RCP2.6 (**b**), 4.5 (**c**) and 8.5 (**d**). The absolute values (km^2^) on the bottom line denote the area of very low/low suitable/moderately suitable/high/very high suitable land. The three diagrams represent the absolute area change (km^2^) for categories of suitability (columns) between the current conditions and future scenarios. The maps were created using ArcGIS Pro v. 2.3.0^46^ (https://www.esri.com/en-us/arcgis/products/arcgis-pro/overview).

As a response to increasing temperature means until 2050 (annual, July, and January), the potential cultivation areas for Burgundy truffles will expand under low- and mid-emission scenarios (~ + 98 km^2^) but decline under RCP8.5 ( ~ − 57 km^2^) (Fig. 4, Table 1). The suitability to cultivate Burgundy truffles in these areas will increase under low-emission scenario (2.6). The actual 8486 km^2^ of high suitability will expand by ~ 3% (250 km^2^). With a negligible ~ 1.5% (39 km^2^) decline of high suitability under mid-emission scenario (4.5), the potential to cultivate Burgundy truffles remains comparable to the current climate conditions (Fig. 4, Table 1). Under the high-emission scenario (8.5), the highly suitable land for Burgundy truffles will decrease only by ~ 7% (572 km^2^). The very highly suitable land will grow from 2 km^2^ by 99–195 km^2^ in 2050 under all scenarios (Fig. 4, Table 1). With an annual rate of change of ~ 4 km^2^ from 2020 to 2050, the cultivation suitability of Burgundy truffles will change much slower compared to Périgord truffles.

**Figure 5:**
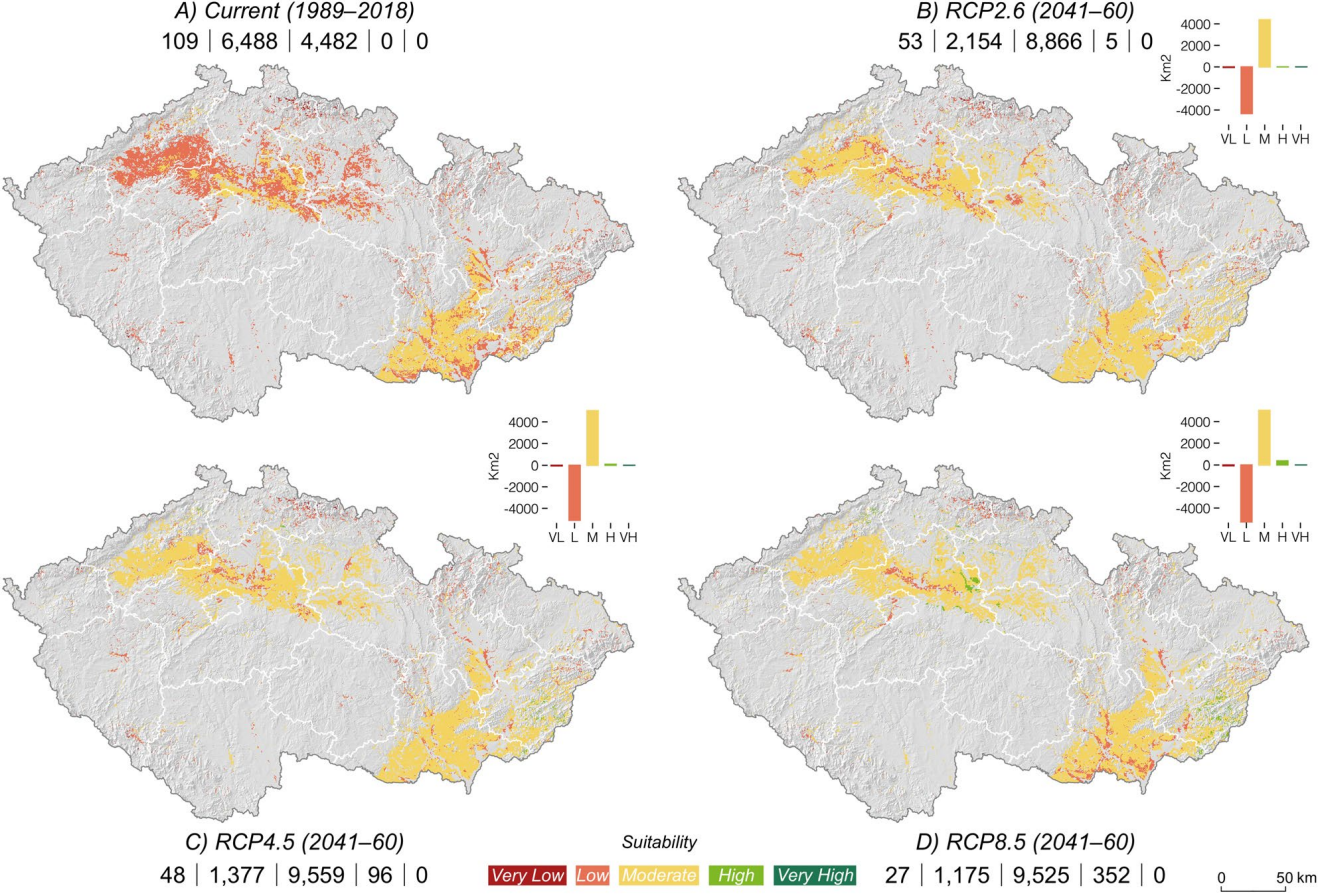
Change of the suitability potential of Périgord truffle areas in Czech Republic between current (**a**; 1989–2018) and future climate conditions described by RCP2.6 (**b**), 4.5 (**c**) and 8.5 (**d**). The absolute values (km^2^) on the bottom line denote the area of very low/low suitable/moderately suitable/high/very high suitable land. The three diagrams represent the absolute area change (km^2^) for categories of suitability (columns) between the current conditions and future scenarios. The maps were created using ArcGIS Pro v. 2.3.0^46^ (https://www.esri.com/en-us/arcgis/products/arcgis-pro/overview).

At a pace of change in the order of 83 km^2^ per year, the suitable land for Périgord truffles will expand nearly 20 times faster than that of Burgundy truffles (Fig. 5, Table 1). While current potential cultivation areas for Périgord truffles will remain constant regardless of future warming, the suitability will rise substantially under all scenarios. The future temperature increase will generate up to 352 km^2^ of high suitability (RCP8.5; above 60%). From its current 4482 km^2^ to ~ 9316 km^2^ (RCP mean), the potential area of moderate suitability to cultivate Périgord truffles will virtually double in 2050 (Fig. 5, Table 1). As a result, the land under very low and low suitability will drop by up to ~ 75% (82 km^2^) and ~ 82% (5313 km^2^) under high-emission scenario.

## Discussion

First, we discuss the uncertainties associated with our modelling experiment. We then outline the ecological and economic implications of a possible increase in central European truffle cultivation under future climate change. Finally, we address issues related to irrigation demands and changes in host tree distribution.

Apart from a few brief reports of past truffle harvests and trade, in tandem with scarce herbarium specimens^54^, the occurrence of truffles in the Czech Republic is basically unknown. It should be further noted that Burgundy truffles are strictly protected, and any harvesting attempt is banned^55^. Moreover, the few written documents from the early and mid twentieth century lack rigorous species identification nor do they provide sufficiently detailed information on the locations of truffle growth. Estimates of the potential truffle occurrence based on the detection of mycorrhiza in the roots of potential host trees were recently provided for the Czech Republic using a PCR screening method^56^. However, we cannot use these data for validation, since species-specific truffle primers for PCR detection are still associated with large uncertainties, and the presence of mycorrhiza alone does not automatically imply the production of fruiting bodies^57^. Supported by numerous Burgundy truffle finds in neighbouring countries^21,58^, our model estimates should be confirmed by extensive field studies.

The predicted increase in cultivation suitability of the Burgundy truffle by 2050 under the low-emission scenario (RCP2.6) indicates that this fungal species will likely benefit from moderate warming. That, in addition to the broad temperature niche^20^ and slower suitability changes, strengthens the notion of climate plasticity of Burgundy truffles and corresponds to the species’ wide geographical distribution across Europe. However, our models suggest that the very strong temperature increase under the high-emission scenario (RCP8.5), together with the cumulative likelihood of summer droughts^27^, will suppress fruit body formation (and thus total production)^20^, regardless of the sporocarp’s ability to survive under extreme aridity. Hence, we expect that the most favourable climate conditions will move northward, which coincides with the predicted latitudinal shift reported in Büntgen et al.^26^, and agrees with the overall climate-induced migration of ectomycorrhiza fungi^1–4^. The lower suitability for the Périgord truffle corresponds with the species’ current restriction to southern Europe^23^. However, the Périgord truffle is expected to benefit largely from climate change, which has already been demonstrated by first signs of the species’ northward migration^24–27^.

Under predicted climate change scenarios, the temperature increase in the Czech Republic will accelerate evapotranspiration, leading to an elevated risk of agricultural drought^59^, which may critically affect truffle cultivation as well^60^. However, with up to 15 years temporal offset between plantation establishment and truffle harvest^51^, irrigation may help to overcome the constraints natural summer precipitation totals will have on truffle growth^61^. Although irrigation systems are currently available on 1.5% of agricultural land in the Czech Republic^62^, artificial water supplies are expected to increase under future climate change^63^. Reducing the limiting effects of evapotranspiration in a warmer world, advanced irrigation techniques may enhance interannual stability and the total production^51^. We therefore expect an increasing potential to cultivate truffles in agricultural regions of southern Moravia and central Bohemia (Figs. 2, 3, 4, 5). Moreover, we speculate that these regions could benefit from an increasing interest in truffle cultivation due to land-use diversification and biodiversity^28^.

Although potential host trees are not specifically included in our modelling experiments, we recognize their importance for evaluating potential truffle habitats. Consequently, we compare the host tree species list from the literature review with the species database from the Nature Conservation Agency of the Czech Republic^64^, and find many examples of common occurrences between the two lists. Despite the fact that most of the cultivated woody vegetation in the Czech Republic is a result of human activities^34^, much of it originates from indigenous species that represent a large portion of the potential truffle host trees. In addition, the wide ecological range of the reported host species is a promising prerequisite for the successful establishment and maintenance of truffle plantations on agricultural land.

We suppose that truffle cultivation efforts under future climate change would be most viable in association with drought-tolerant oaks (*Quercus* spp.) (Fig. 1), which used to grow naturally in many of the country’s low-elevation regions^34^. Truffles will most likely benefit from the occurrence of *Quercus pubescens* that is already expanding from the southernmost part of the Czech Republic^34^, and *Quercus cerris*, which is naturally distributed across Pannonia, the Balkan, and the Apennine^65^.

## Conclusions

Based on a comprehensive literature review, we estimate the theoretically viable ecological ranges for Burgundy and Périgord truffles, and use this information in ecological niche models to predict the species-specific truffle cultivation potential in the Czech Republic under current and future climate conditions in 2020 and 2050, respectively. Although associated with uncertainties, climate change will most likely facilitate the cultivation of both truffle species on alkaline soils of pH > 7, generating a wide range of ecological and economic benefits.

## Supplementary information

Supplementary Information
