# Supplementary material for: Predicted climate change will increase the truffle cultivation potential in central Europe
Source: Sci Rep. 2020 Dec 4;10:21281. doi: 10.1038/s41598-020-76177-0 (PMC7719165; doi:10.1038/s41598-020-76177-0)
Supplement: Supplementary file 1 — Supplementary Information [file 41598_2020_76177_MOESM1_ESM.pdf]

Supporting Information: Predicted climate change will increase the truffle cultivation potential in central Europe

Tomáš Čejka, Miroslav Trnka, Paul J. Krusic, Ulrich Stobbe, Daniel Oliach, Tomáš Václavík, Willy Tegel, and Ulf Büntgen

Supplementary Table S1. The pH parameters of the major soil units (agricultural land) and forest typological units (non-agricultural). All pH values that are located within the unit of interest are used to calculate its average pH, standard deviation, and median.

Major Soil Unit	Average pH	Standard Deviation	Median pH
1	7.22	0.39	7.40
2	6.83	0.43	6.80
3	7.02	0.45	7.20
4	7.00	0.46	7.10
5	7.08	0.44	7.20
6	7.16	0.42	7.30
7	7.20	0.40	7.40
8	7.25	0.42	7.40
9	6.71	0.41	6.70
10	6.83	0.44	6.80
11	6.60	0.40	6.50
12	6.67	0.43	6.60
13	6.77	0.46	6.70
14	6.58	0.38	6.50
15	6.50	0.37	6.40
16	6.81	0.43	6.80
17	6.89	0.42	7.00
18	6.82	0.45	6.80
19	7.18	0.41	7.30
20	7.04	0.44	7.20
21	6.91	0.45	7.00
22	6.88	0.48	6.90
23	6.85	0.45	6.90
24	6.81	0.45	6.80
25	6.52	0.38	6.40
26	6.51	0.37	6.40
27	6.62	0.44	6.50
28	6.71	0.46	6.60
29	6.46	0.36	6.40
30	6.67	0.44	6.60
31	6.77	0.46	6.70
32	6.47	0.36	6.40
33	6.75	0.43	6.70
34	6.36	0.27	6.30

35	6.40	0.30	6.30
36	6.42	0.33	6.30
37	6.61	0.45	6.50
38	6.57	0.40	6.50
39	6.78	0.45	6.70
40	6.92	0.55	7.00
41	7.18	0.49	7.40
42	6.58	0.38	6.50
43	6.50	0.34	6.40
44	6.48	0.33	6.40
45	6.51	0.35	6.40
46	6.47	0.34	6.40
47	6.46	0.34	6.40
48	6.47	0.34	6.40
49	6.67	0.42	6.60
50	6.42	0.31	6.30
51	6.63	0.40	6.60
52	6.49	0.33	6.40
53	6.53	0.37	6.40
54	6.81	0.47	6.80
55	6.97	0.46	7.10
56	6.90	0.45	7.00
57	6.97	0.44	7.10
58	6.79	0.46	6.80
59	6.83	0.46	6.80
60	7.17	0.43	7.30
61	7.14	0.41	7.30
62	7.07	0.45	7.20
63	7.00	0.44	7.10
64	6.52	0.38	6.40
65	6.82	0.50	6.80
66	6.68	0.44	6.50
67	6.46	0.34	6.40
68	6.53	0.40	6.40
69	6.49	0.43	6.30
70	6.81	0.45	6.80
71	6.45	0.35	6.30
72	6.67	0.50	6.55
73	6.48	0.31	6.40
74	6.50	0.27	6.40
75	6.69	0.54	6.70
76	6.55	0.35	6.55
77	6.93	0.60	7.00
78	no data	no data	no data
79	forest area	forest area	forest area
Forest Typological Unit	Average pH	Standard Deviation	Median pH
0C	6.60	0.77	6.40
1C	6.00	only one value	6.00

1D	6.12	0.32	6.12
1H	7.36	0.85	7.50
1L	6.16	0.80	6.19
1S	6.08	0.58	6.00
1V	7.11	only one value	7.11
1W	6.82	0.61	6.70
1X	5.10	only one value	5.10
1Z	6.30	only one value	6.30
2A	6.88	0.51	6.70
2B	6.56	0.54	6.50
2C	6.20	0.29	6.20
2D	5.51	only one value	5.51
2H	6.14	0.87	6.05
2I	5.99	0.52	6.01
2K	6.20	only one value	6.20
2L	7.74	0.37	7.74
2S	6.54	0.64	6.50
2W	6.56	0.39	6.40
3A	5.83	only one value	5.83
3B	6.16	0.21	6.10
3D	5.86	0.36	6.00
3H	6.41	0.46	6.40
3J	5.17	only one value	5.17
3K	6.32	0.29	6.30
3O	6.18	0.22	6.10
3S	6.67	0.84	6.60
3U	6.65	1.89	6.65
3V	5.30	only one value	5.30
4B	6.23	0.62	6.10
4D	6.10	0.14	6.10
4O	6.00	only one value	6.00
4S	6.40	only one value	6.40
4W	7.35	0.48	7.50
5B	5.27	only one value	5.27
5D	5.28	only one value	5.28
5K	6.20	only one value	6.20
6A	6.20	only one value	6.20
6K	6.09	0.56	6.25
6P	6.70	only one value	6.70
6S	6.27	0.31	6.20
7K	6.67	0.67	6.50
7S	6.45	0.21	6.45
8T	6.30	only one value	6.30

Supplementary Table S2. The review of the ecological requirements of Burgundy and Périgord truffle based on data from the international (English/Spanish/French/Italian) peer reviewed journal papers, reports and one monography (since 1981). When appended by an asterisk, the climate data are extrapolated from the CRU TS database (ver. 4.03, 0.5 × 0.5°, 1981–2010)[58] using the coordinates/location (where available, as some locations are kept in secrecy). If the original research provides only the data range and/or several sites, we included the average to simplify the inputs. The host tree species is presented only if the species is currently growing in the Czech Republic.

**Burgundy truffle (*Tuber aestivum*)**										
Country	Annual Temperature (°C)	July Temperature (°C)	January Temperature (°C)	Annual Precipitation (mm)	Summer Precipitation (mm)	Average pH	Elevation (m)	Host Trees	Short Description	Reference Number
Hungary	10.20	21.50*	0.90*	570	185*	7.55	90	*Q. cerris, Q. robur*	Burgundy truffle plantation near Jászivány village (47.5°N, 20.2°E).	1
Greece	–						300	*Q. pubescens*	Naturally occurring Burgundy truffles in northern Greece (150–450 m a.s.l.). Exact locations are not specified.	2
Hungary	10.50	21.30*	1.00*	555	182*	7.55	150	*Q. cerris, Q. robur*	Three Burgundy truffle plantations and 20 natural habitats in Jászság region (47.5°N, 19.92°E).	3
Poland	8.00	–		600	–	7.27	230	*C. betulus, C. avellana, F. sylvatica, Q. robur, T. cordata*	Five Burgundy truffle natural habitats in Central Poland, not further specified.	4
Poland	8.00	18.80*	-3.00*	550	202*	7.60	200	*C. avellana, Q. robur*	One Burgundy truffle plantation in eastern Poland (51.15°N, 23.48°E).	5
Poland	8.00	19.10*	-2.10*	600	262*	7.14	280	*C. avellana, F. sylcatica, P. nigra, Q. robur, T. cordata*	Four Burgundy truffle natural habitats (50.27°N, 20.34°E).	6
France	10.33	18.70	2.70	884	–	7.6	880	*Coryllus, Quercus, Tilia spp.*	A 25 samples, not further specified.	7
Morocco	15.10*	25.80*	8.00*	434*	21*	7.50	1,350	–	Five Burgundy truffle natural habitats Central Middle Atlas (33.56°N, 4.76°W).	8
Poland	9.10*	18.40*	-1.80*	616*	237*	–	230	*F. sylvatica, Q. petraea, Q. robur*	Five Burgundy truffle natural habitats in Łódź region (50.76°N, 19.41°E).	9
Italy	12.75	–		874	–	7.75	675	*Q. petraea*	Average of two Burgundy truffle locations in Teramo and Piacenza. Study is based on soil (brûlés) samples.	10(a)
Spain	9.70	–		797	–	7.75	1,000	–	Soil (brûlés) samples from Guadalajara region.	10(b)
France	10.40*	19.00*	1.80*	732	211*	7.90	335	*C. avellana*	One Burgundy truffle plantation near Daix (47.35°N, 4.50°E).	11
Switzerland, Germany	6.80	–				7.07	532	*C. betulus, F. sylvatica, O. carpinifolia*	Sixteen truffle locations in Switzerland and Germany.	12
Italy	10.60	21.00*	1.90*	810	211*	8.06	721	*Q. pubescens*	Nine Burgundy truffle locations in Ponte dell'Oglio region (44.87°N, 9.64°E). Study is based on soil (brûlés) samples.	13
Romania	–					7.11	717	*Quercus spp.*	Review of soil parameters and distribution modelling of Burgundy truffles in Subcarpathian Hills.	14

Italy	9.50	19.40*	1.70*	950	162*	7.85	1,000	*Q. cerris, Q. pubescens, P. nigra*	One Burgundy truffle plantation near Chiusi della Verna (43.70°N, 11.94°E).	15
Germany	8.67	10.50	7.10	880	–		541	*C. betulus, F. sylvatica, P. abies, Q. robur, Tilia spp.*	A 116 truffle locations in southern Germany.	16
–	9.03	19.36	1.33	755	–	7.41	470	*C. betulus, C. avellana, F. sylvatica, Q. cerris, Q. petraea, Q. robur*	Review on ecological factors of truffles.	17
Germany	6.45	15.40	-2.15	873	–	7.62	716	*F. sylvatica, P. abies, Q. robur*	Two Burgundy truffle locations in southwestern Germany.	18
Sweden	6.84	16.30	-1.80	528	183*	7.47	41	*C. avellana, Q. robur*	Eighteen Burgundy truffle locations on Gotland (57.50°N, 18.50°E).	19
Sweden	6.84	16.30	-1.80	528	183*	7.57	41	*C. betulus, C. avellana, Q. robur*	Twenty-four Burgundy truffle plantations on Gotland (57.50°N, 18.50°E).	20
France	10.50*	19.00*	1.80*	940	224*	–	360	*C. avellana*	Burgundy truffle location near Rollainville (48.36°N, 5.74°E). The results are provided by Christoper Robin – the main author of the chapter on Burgundy truffle in Zambonelli et al. (2016). Other localities mentioned in the publication were not published and are therefore not included here.	21
Turkey	12.80*	26.40	6.70	568	51*	7.50	1,200	*P. nigra*	Burgundy truffle sites in Denizli, Turkey	22
Sicily (Italy)	16.71*	24.86*	9.89*	554*	28*	–	830	–	Distribution of Burgundy truffle in Sicily.	23, 24
Spain	10.80	20.62	3.09	650	70	–	1,225	*P. sylvestris, P. nigra*	A total of 145 Burgundy truffle brûlés in central Spain.	25
Italy	11.6*	21.20*	3.40*	777*	128*	–	900	*Q. cerris*	Six fruiting bodies of Burgundy truffle in Molise region, southern Italy.	26
Italy	12.7*	22.30*	4.5*	799*	140*	–	350	*Q. pubescens, C. avellana, O. carpinifolia*	Burgundy truffle orchard In Spoleto, central Italy.	27
Average	10.0768000	19.7733333	2.0552381	701.0000000	157.6470588	7.5405263	569.0370370	*C. betulus, C. avellana, F. sylvatica, O. carpinifolia, P. abies, P. nigra, Q. cerris, Q. petraea, Q. pubescens, Q. robur, T. cordata, P. sylvestris*		
Standard Deviation	2.5029706	3.5000317	3.5278992	150.8959354	71.7929549	0.2577938	375.4031395			
**Périgord truffle (*Tuber melanosporum*)**										
Italy	–					7.70	–		Comparison of ecological requirements between Périgord and White truffle in Central Italy.	28
Italy	–					8.00	–		Study of Périgord truffle soils and flora in Central Italy.	29
France	14.00	23.50	–						Effect of subsoil structural characteristics on production of Périgord truffle.	30
France	–					8.08	–		Application of PCA on the Périgord truffle soil characteristics.	31
France	–					7.75	–		Study of mycorrhization of Périgord truffle based on physical-chemical soil characteristics.	32
France	–					7.75	–		Soil mechanics and mycorrhizae development of Périgord truffle.	33
Spain	–					7.64	–		Analysis of the productivity and ecological characterization of *Quercus faginea* Lam. as a host tree of Périgord truffle.	34
Spain	9.70	18.30	1.40	–					Relationship between climate and productivity of Périgord truffle in Alto Tajo (Guadalajara y Cuenca).	35

Spain	9.70	19.81*	2.25*	797	99*	7.89	1,250	–	Twenty soil sites (brûlés) of Périgord truffle in Guadalajara region (40.60°N, 1.90°W).	36
Spain	11.00	20.23*	2.14*	500	102*	8.29	971	–	Three Périgord truffle plantations near Sarrión, Teruel (40.15°N, 0.84°W).	37
New Zealand	–			1,086	–	7.90	–		Potential of Périgord truffle on acidic and amended soils in New Zealand.	38
Spain	11.00	–		550	41	–			Overview of the Périgord truffle research.	39
France, Spain	–					7.73	–		Four clusters of truffle natural habitats and plantations.	40
France	9.50	18.99*	1.71*	941	224*	7.97	360	*C. avellana*	Analysis of the carbon transfer to Périgord truffles in plantations in western Vosges (48.31°N, 5.74°E).	41
Italy	11.30	22.33*	4.47*	983	141*	7.85	740	*Q. pubescens*	One truffle plantation in Central Apennines (42.98°N, 12.87°E).	42
France	13.45*	21.91*	5.67*	720*	149*	8.00	150	*Q. pubescens*	Soil samples (brûlés) in nine Périgord truffle plantations near Cahors.	43
Spain	12.20	18.76*	2.90*	744	161*	8.28	625	–	Four Périgord truffle plantations in Tierra Estella.	44(a)
Spain	12.20	18.76*	2.90*	744	161*	8.40	680	–	One natural Périgord truffle habitat in Tierra Estella.	44(b)
Spain	12.40	20.39*	4.29*	773	151*	8.10	580	*C. avellana*	Three Périgord truffle plantations in Valdorba.	44(c)
France	–					7.90	–		Interaction between pH and Périgord truffle mycelium.	45
Italy	–					7.90	–		Overview on Périgord truffle soils in Rieti province.	46
Italy	–					7.95	–		Overview on soils of naturally occurring Périgord truffles, central Apennines.	47
Spain	11.70	20.30	5.25	664	128	–			Review of the truffle cultivation in Spain.	48
–	–	19.25	4.50	1,050	–				Monography about the trufficulture.	49
Spain	14.54*	24.51*	5.83*	750	88*	8.50	–		Distribution map of Périgord truffle in Zaragoza province employing several climatic variables.	50
France	11.13*	19.51*	3.28*	671*	170*	7.75	200	–	One plantation of Périgord truffles near Rognes in Provence (48.83°N, 3.58°E).	51
France	–					8.12	–		Monography about trufficulture.	52
–	12.90	20.60	5.80	777	193	–			Review of the climatic parameters of Périgord truffle.	53
France	12.55*	21.41*	4.78*	807*	150*	8.5	–		Eight truffle sites/markets in France. Published also in chapter by Francois Le Tacon in Zambonelli et al. (2016)	54, 55
Spain	11.10	20.50	3.50	646	63	–			Agroclimatic zoning of the truffle wild stands based on >100 sites.	56
–								*C. betulus, P. nigra, T. cordata*	Review of host tree species; chapter by Milan Gryndler in Zambonelli et al. (2016)	57
Average	11.7864706	20.5329412	3.7918750	776.6470588	134.7333333	7.9978261	617.3333333	*C. avellana, Q. pubescens, C. betulus, P. nigra, T. cordata*		
Standard Deviation	1.4164659	1.6725421	1.4388633	156.4751216	47.0722376	0.2468225	334.0928015			

Supplementary Table S3. Environmental requirements (parameters) of Burgundy truffle and Périgord truffle that are used to compute the potentially suitable area. The weight of each requirement is based on the rank sum method[59], following the expert judgment of literature evidence. pH = H_2O-detected pH level, MAT = Mean annual temperature (°C), TSP = Total summer precipitation (mm), TAP = Total annual precipitation (mm), MJUT = Mean July temperature (°C), MJAT = Mean January temperature (°C), E = elevation (m).

Parameter	Rank (Importance)	Weighted Score	Truffle Species	Suitability Classes of Ecological Ranges (% Probability)				
				5 (100–81)	4 (80–61)	3 (60–41)	2 (40–21)	1 (20–0.5)
pH (H_2O)	1	0.1842	Burgundy<br>Périgord	>7.39	7.39–7.30	7.29–7.20	7.19–7.10	7.09–7.00
MAT	2	0.1579	Burgundy	9.45–10.71	9.44–8.77<br>10.72–11.39	8.76–7.98<br>11.40–12.18	7.97–6.88<br>12.19–13.28	6.87–3.64<br>13.29–16.52
			Périgord	11.44–12.15	11.43–11.05<br>12.16–12.53	11.04–10.60<br>12.54–12.98	10.59–9.98<br>12.99–13.60	9.97–8.15<br>13.61–15.44
TAP	2	0.1579	Burgundy	664–739	663–623<br>740–780	622–575<br>781–828	574–509<br>829–894	508–313<br>895–1,090
			Périgord	738–816	737–696<br>817–859	695–646<br>860–908	645–577<br>909–977	576–375<br>978–1,180
MJAT	3	0.1316	Burgundy	1.17–2.95	1.16–0.22<br>2.96–3.91	0.21– -0.90<br>3.92–5.02	-0.91– -2.46<br>5.03–6.58	-2.47– -7.02<br>6.59–11.14
			Périgord	3.44–4.16	3.43–3.05<br>4.17–4.55	3.04–2.59<br>4.56–5.00	2.58–1.96<br>5.01–5.64	1.95–0.10<br>5.65–7.50
MJUT	3	0.1316	Burgundy	18.90–20.66	18.89–17.95<br>20.67–21.61	17.94–16.84<br>21.62–22.72	16.83–15.30<br>22.73–24.26	15.29–10.77<br>24.27–28.79
			Périgord	20.12–20.96	20.11–19.67<br>20.97–21.41	19.66–19.14<br>21.42–21.94	19.13–18.40<br>21.95–22.68	18.39–16.23<br>22.69–24.84
TSP	3	0.1316	Burgundy	140–176	139–121<br>177–195	120–98<br>196–218	97–67<br>219–250	66– -26<br>251–343
			Périgord	124–147	123–111<br>148–159	110–96<br>160–174	95–75<br>175–195	74–14<br>196–256
E	4	0.1053	Burgundy	475–664	474–373<br>665–766	372–254<br>767–885	253–89<br>886–1,050	88– -397<br>1,051–1,536
			Périgord	534–702	533–443<br>703–793	442–337<br>794–899	336–190<br>900–1,045	189– -242<br>1,046–1,478

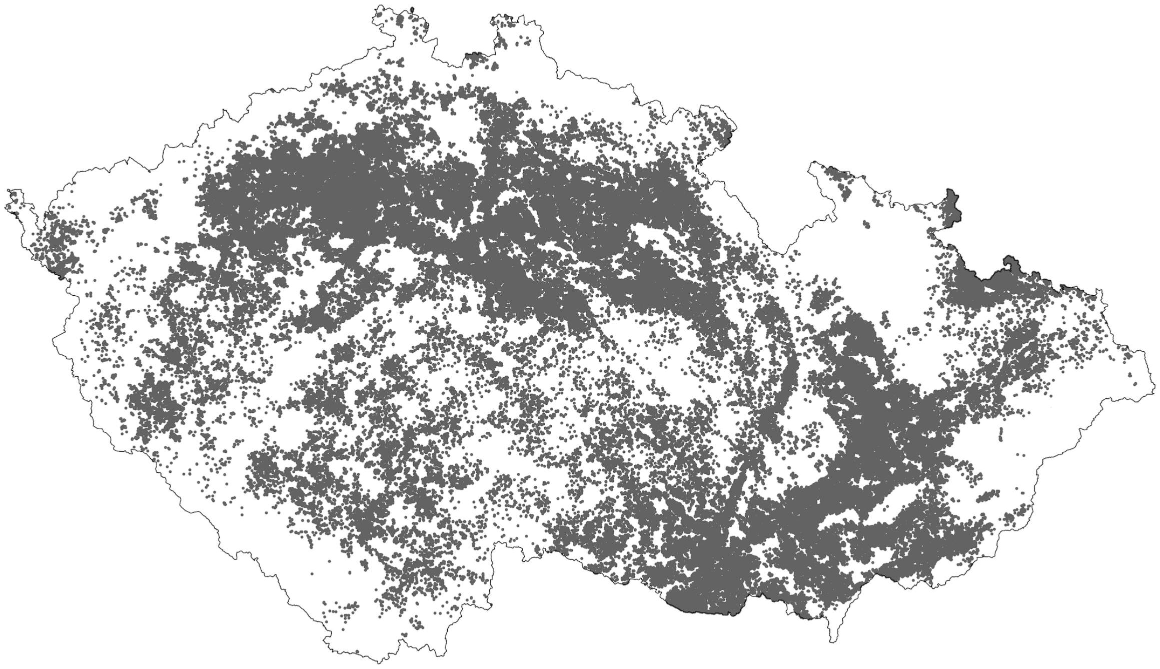

Supplementary Figure 1. Distribution of the original pH dataset containing more than 150,000 field measurements (grey points). Data coverage corresponds to 0.48 pH field measurements per one 500m cell. The map was created using ArcGIS Pro v. 2.3.0 [60] (https://www.esri.com/en-us/arcgis/products/arcgis-pro/overview).

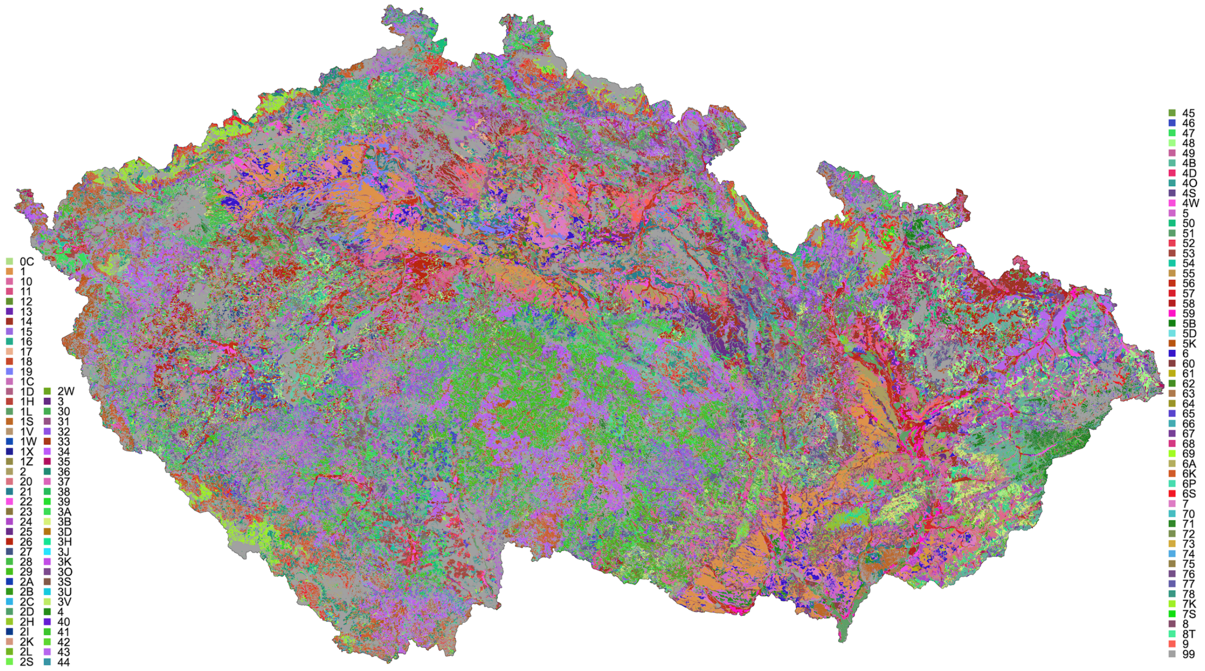

Supplementary Figure 2. Distribution of typological soil units in Czech Republic. The map was created using ArcGIS Pro v. 2.3.0 [60] (https://www.esri.com/en-us/arcgis/products/arcgis-pro/overview).

Supplementary References

1. Büntgen, U. *et al.* New Insights into the Complex Relationship between Weight and Maturity of Burgundy Truffles (Tuber aestivum). *Plos ONE* **12,** e0170375; 10.1371/journal.pone.0170375 (2017).
2. Diamandis, S. & Perlerou, C. Recent records of hypogeous fungi in Greece. *Acta Mycol.* **43,** 139–142 (2008).
3. Gógán, A. C., Nagy, Z., Dégi, Z., Bagi, I. & Dimény, J. Ecological characteristics of a Hungarian summer truffle (*Tuber aestivum* Vittad.) producing area. *Acta Mycol.* **47,** 133–138; 10.5586/am.2012.015 (2012).
4. Hilszczańska, D., Sierota, Z. & Palenzona, M. New *Tuber* species found in Poland. *Mycorrhiza* **18,** 223–226; 10.1007/s00572-008-0175-4 (2008).
5. Hilszczańska, D., Szmidla, H., Horak, J. & Rosa-Gruszecka, A. Ectomycorrhizal communities in a *Tuber aestivum* Vittad. orchard in Poland. *Open Life Sci.* **11,** 348–357; 10.1515/biol-2016-0046 (2016).
6. Hilszczańska, D., Szmidla, H., Sikora, K. & Rosa-Gruszecka, A. Soil Properties Conducive to the Formation of *Tuber aestivum* Vitt. Fruiting Bodies. *Pol. J. Environ. Stud.* **28,** 1713–1718; 10.15244/pjoes/89588 (2019).
7. Le Tacon, F. Les sols du Bassin Parisien susceptibles de convenir aux truffières à *Tuber uncinatum*. in *La Truffe de Bourgogne: histoire, biologie, écologie, culture, récolte, gastronomie* (eds. Chevalier, G. & Frochot, H.) 84–101 (Pétrarque, 1997)
8. Khabar, L. Contribution to the study of the ecology of *Tuber aestivum* in Morocco: phytosociological and geological aspects. *Österr. Z. Pilzk.* **19,** 261–264. (2010).
9. Ławrynowicz, M., Krzyszczyk, T. & Fałdziński, M. Occurrence of black truffles in Poland. *Acta Mycol.* **43,** 143–151 (2008).

10. Menta, C. *et al.* Does the natural "microcosm" created by *Tuber aestivum* affect soil microarthropods? A new hypothesis based on Collembola in truffle culture. *Appl. Soil Ecol.* **84,** 31–37; 10.1016/j.apsoil.2014.06.012 (2014).

11. Molinier, V. *et al.* First identification of polymorphic microsatellite markers in the Burgundy truffle, *Tuber aestivum* (*Tuberaceae*). *Appl. Plant Sci.* **1,** 1200220; 10.3732/apps.1200220 (2013).

12. Moser, B. *et al.* Ecological indicators of *Tuber aestivum* habitats in temperate European beech forests. *Fungal Ecol.* **29,** 59–66; 10.1016/j.funeco.2017.06.002 (2017).

13. Pinto, S., Gatti, F., García-Montero, L. G. & Menta, C. Does soil fauna like truffles just as humans do? One-year study of biodiversity in natural brûlés of *Tuber aestivum* Vittad. *Sci. Total Environ.* **584–585,** 1175–1184; 10.1016/j.scitotenv.2017.01.181 (2017).

14. Păcurar, H. *et al.* Identification of Soils Factors Influence in the Distributions of *Tuber aestivum* in Transylvanian Subcarpathian Hills, Romania. *Not. Bot. Horti. Agrobo.* **47,** 478–486; 10.15835/nbha47111378 (2019).

15. Salerni, E., D'Aguanno, M., Leonardi, P. & Perini, C. Ectomycorrhizal communities above and below ground and truffle productivity in a *Tuber aestivum* orchard. *Forest Sys.* **23,** 329–338; 10.5424/fs/2014232-04777 (2014).

16. Stobbe, U. *et al.* Spatial distribution and ecological variation of re-discovered German truffle habitats. *Fungal Ecol.* **5,** 591–599; 10.1016/j.funeco.2012.02.001 (2012).

17. Stobbe, U. *et al.* Potential and limitations of Burgundy truffle cultivation. *Appl. Microbiol. Biotechnol.* **97,** 5215–5224; 10.1007/s00253-013-4956-0 (2013a).

18. Stobbe, U. *et al.* New evidence for the symbiosis between *Tuber aestivum* and *Picea abies*. *Mycorrhiza* **23,** 669–673; 10.1007/s00572-013-0508-9 (2013b).

19. Wedén, C., Chevalier, G. & Danell, E. *Tuber aestivum* (syn. *T. uncinatum*) biotopes and their history on Gotland, Sweden. *Mycol. Res.* **108,** 304–310; 10.1017/s0953756204009256 (2004).
20. Wedén, C., Pettersson, L. & Danell, E. Truffle cultivation in Sweden: Results from *Quercus robur* and *Coryllus avellana* field trials on the island of Gotland. *Scand. J. For. Res.* **24,** 37–53; 10.1080/02827580802562056 (2009).
21. Robin, C. *et al.* Soil Characteristics for *Tuber aestivum* (Syn. *T. uncinatum*). in *True Truffle (Tuber spp.) in the World* (eds. Zambonelli, A., Iotti, M. & Murat, C.) 211–233 (Springer International Publishing, 2016).
22. Gezer, K, Kaygusuz, O., Çelik, A. & Işiloğlu, M. Ecological characteristics of truffles growing in Denizli Province, Turkey. *J. Food Agric. Environ.* **12,** 1105–1109; 10.1234/4.2014.5296 (2014).
23. Venturella, G., Saitta, A., Sarasini, M., Montecchi, A. & Gori, L. Contribution to the knowledge of hypogeous fungi from Sicily (S-Italy). *Fl. Medit.* **14,** 275–284 (2004).
24. Venturella, G., Pecorella, E., Saitta, A., Zambonelli, A. & Morarra, M. Ecology and distribution of hypogeous fungi from Sicily (southern Italy). *Cryptogamie Mycol.* **27,** 201–217 (2006).
25. Garcia-Montero, L. G., Moreno, D., Monleon, V. & Arredondo-Ruiz, F. Natural production of *Tuber aestivum* in central Spain: *Pinus* spp. *versus Quercus* spp. brûlés. *Forest Syst.* **23,** 394–399; 10.5424/fs/2014232-05112 (2014).
26. Monaco, P. *et al.* The bacterial communities of *Tuber aestivum*: preliminary investigations in Molise region, Southern Italy. *Ann. Microbiol.* **70,** 1–10; 10.1186/s13213-020-01586-5 (2020).

27. Benucci, G. M. N. *et al.* Ectomycorrhizal communities in a productive *Tuber aestivum* Vittad. orchard: composition, host influence and species replacement. *FEMS Microbiol. Ecol.* **76,** 170–184; 10.1111/j.1574-6941.2010.01039.x (2011).

28. Bencivenga, M. & Granetti, B. Ricerca comparativa sulle esigenze ecologiche di *Tuber magantum* Pico e *Tuber melanosporum* Vitt. dell'Italia centrale. *Annali della Facoltà di Agraria, Università degli studi di Perugia* **42,** 861–872 (1988).

29. Bencivenga, M., Calandra, R. & Granetti, B. Ricerche sui terreni e sulla flora delle tartufaie naturali di *T. melanosporum* Vitt. dell'Italia centrale. *Atti del Secondo Congresso Internazionale sul Tartufo, Spoleto* **24,** 337–374 (1990).

30. Callot, G. & Jaillard, B. Effect of structural characteristics of subsoil on the fruiting of *Tuber melanosporum* and other mycorrhizal fungi. *Agronomie* **16,** 405–419; 10.1051/agro:19960701 (1996).

31. Delmas, J., Brian, C., Delpech, P. & Soyer, J. P. Application del'analyse en composantes principales à une tentative de caractérisation physicochimique des sols trufficoles français. *Mushroom Sci.* **11,** 855–867 (1981a).

32. Delmas, J., Chevalier, G., Villenave, P. & Bardet, M. C. Étude de la mycorhization par *Tuber melanosporum* en fonction des caracteristiques physico-chimiques et mecaniques. *INRA – Le centre technique au service de la filière fruits et legumes* **12,** 1–7 (1981b).

33. Delmas, J., Chevalier, G., Villenave, P. & Bardett, M. C. Mécaniques des sols et mycorhizes de *Tuber melanosporum*. *Les Colloques De L'inra* **13,** 329–335 (1982).

34. García-Montero, L. G., Manjón, J. L. & Casermeiro, M. A. Análisis productivo y caracterización ecológica primaria de *Quercus faginea* Lam. como simbionte de *Tuber melanosporum* Vitt. *Actes du Ve Congrès International, Science et culture de la truffe, Aix-en-Provence*, 4209–4213 (2001).

35. García-Montero, L.G., Moreno, A., Pascual, C. & Manjón, J. L. Evaluación del clima en la producción trufera (trufa negra: *Tuber melanosporum* Vitt.) del Alto Tajo (Guadalajara y Cuenca). *Revista Forestal Española* **31,** 23–29 (2002).

36. García-Montero, L. G., Valverde-Asenjo, I., Díaz, P. & Pascual, C. Statistical patterns of carbonates and total organic carbon on soils of *Tuber rufum* and *T. melanosporum* (black truffle) brûlés. *Aust. J. Soil. Res.* **47,** 206–212; 10.1071/SR08084 (2009).

37. García-Montero, L. G., Valverde-Asenjo, I., Grande-Ortíz, M. A., Menta, C. & Hernando, I. Impact of earthworm casts on soil pH and calcium carbonate in black truffle burns. *Agrofor. Syst.* **87,** 815–826; 10.1007/s10457-013-9598-9 (2013).

38. Hall, I. R. & Wang, Y. Culture de la truffe noire du Périgord en Nouvelle-Zélande sur sols naturellement acides, amendes. *Resumes des interventions, Journee nationale de la trufficulture, Paris,* 3–8. (2003).

39. Hernández, A. *Líneas de investigación sobre trufa: Actas de las I Jordanas Internacionales de Trufficultura.* (Asopiva, 1994).

40. Jaillard, B. *et al.* Alkalinity and structure of soils determine the truffle production in the Pyrenean Regions. *Forest Syst.* **23,** 364–377; 10.5424/fs/2014232-04933 (2014).

41. Le Tacon, F. *et al.* Carbon Transfer from the Host to *Tuber melanosporum* Mycorrhizas and Ascocarps Followed Using a ^{13}C Pulse- Labeling Technique. *PLOS One* **8,** e64626; 10.1371/journal.pone.0064626 (2013).

42. Lulli, L., Bragato, G. & Gardin, L. Occurrence of Tuber melanosporum in relation to soil surface layer properties and soil differentiation. *Plant Soil* **214,** 85–92; 10.1023/A:1004602519974 (1999).

43. Napoli, C. *et al.* Tuber melanosporum, when dominant, affects fungal dynamics in truffle grounds. *New Phytol.* **185,** 237–247; 10.1111/j.1469-8137.2009.03053.x. (2010).

44. Parladé, J., De la Varga, H., De Miguel, A. M., Sáez, R. & Pera, J. Quantification of extraradical mycelium of Tuber melanosporum in soils from truffle orchards in northern Spain. *Mycorrhiza* **23,** 99–106; 10.1007/s00572-012-0454-y. (2013).

45. Poitou, N., Villennave, P., Baudet, D. & Delmas, J. Croissance in vitro du mycélium de Tuber melanosporum Vitt. et de certains compétiteurs en fonction du pH du milieu. *Comptes Rendus des Seances de l'Académie d'Agriculture de France* **69,** 1363–1369 (1983).

46. Raglione, M., Lorenzoni, P., De Simone, C., Monaco, R. & Angius, A. Osservazioni sulle caratteristiche pedologiche di alcuni siti di tartufo nero pregiato (*Tuber melanosporum*) in provincia di Rieti. *Micologia e vegetazione mediterranea* **7,** 211–224 (1992).

47. Raglione, M., Spadoni, M., Cavelli, S., Lorenzoni, P. & De Simone, C. Les sols des truffieres naturelles de *Tuber melanosporum* Vitt. dans l'Apennin Central (Italia). *Actes du Ve Congrès International, Science et culture de la truffe, Aix-en-Provence*, 5276–5280 (2001).

48. Reyna, S. *La trufa, truficultura y selvicultura trufera.* (Mundi-Prensa, 2000).

49. Ricard, J. M. *La truffe: Guide technique de trufficulture.* (Centre Technique Interprofessionnel des Fruits et Légumes, 2003).

50. Serrano-Notivoli, R., Martín-Santafé, M., Sánchez, S. & Barriuso, J. J. Cultivation potentiality of black truffle in Zaragoza province (Northeast Spain). *J. Maps* **12,** 994–998; 10.1080/17445647.2015.1113392 (2016).

51. Shaw, P. J. A., Lankey, K. & Jourdan, A. Factors affecting yield of *Tuber melanosporum* in a *Quercus ilex* plantation in southern France. *Mycol. Res.* **100,** 1176–1178; 10.1016/S0953-7562(96)80177-8 (1996).

52. Sourzat, P. *Guide pratique de trufficulture.* (Lycée professionnel agricole et viticole de Cahors, 1997).

53. Thomas, P. W. An analysis of the climatic parameters needed for *Tuber melanosporum* cultivation incorporating data from six continents. *Mycosphere* **5,** 137–142; 10.5943/mycosphere/5/1/5 (2014).

54. Le Tacon, F. *et al.* Climatic variations explain annual fluctuations in French Périgord black truffle wholesale markets but do not explain the decrease in black truffle production over the last 48 years. *Mycorrhiza*. **24 (Suppl 1),** S115–S125; 10.1007/s00572-014-0568-5 (2014).

55. Le Tacon, F. Influence of Climate on Natural Distribution of *Tuber* species and Truffle Production. in *True Truffle (Tuber spp.) in the World* (eds. Zambonelli, A., Iotti, M. & Murat, C.) 153–169 (Springer International Publishing, 2016).

56. Garcia-Barreda, S., Sánchez, S., Marco, P. & Serrano-Notivoli, R. Agro-climatic zoning of Spanish forests naturally producing black truffle. *Agric. For. Meteorol.* **269–270,** 231–238; 10.1016/j.agrformet.2019.02.020 (2019).

57. Gryndler, M. True Truffle Host Diversity. in *True Truffle (Tuber spp.) in the World* (eds. Zambonelli, A., Iotti, M. & Murat, C.) 267–283 (Springer International Publishing, 2016).

58. Harris, I., Jones, P. D., Osborn, T. J. & Lister, D. H. Updated high-resolution grids of monthly climatic observations – the CRU TS3.10 Dataset. *Int. J. Climatol.* **34,** 623–642; 10.1002/joc.3711 (2014).

59. Malczewski, J. *GIS and multicriteria decision analysis.* (John Wiley, 1999).

60. ESRI. *ArcGIS Pro: Release 2.3.0.* (Environmental Systems Research Institute, 2019).
